# A Hybrid Security Framework for Train-to-Ground (T2G) Communication Using DOA-Optimized BPNN Detection, Bayesian Risk Scoring, and RL-Based Response

**DOI:** 10.3390/s25103208

**Published:** 2025-05-20

**Authors:** Chaoyuan Sun, Weijiao Zhang, Peng Sun, Hui Wang, Chunhui Yang

**Affiliations:** 1 Postgraduate Department, China Academy of Railway Sciences, Beijing 100081, China; scydzh@163.com; 2 Institute of Computing Technology, China Academy of Railway Sciences Corporation Limited, Beijing 100081, China

**Keywords:** railway cybersecurity, intrusion detection system, Dream Optimization Algorithm, Bayesian risk scoring, reinforcement learning, intelligent security defense framework, Train-to-Ground communication system

## Abstract

With the widespread adoption of wireless communication technologies in modern high-speed rail systems, the Train-to-Ground (T2G) communication system for Electric/Diesel Multiple Units (EMU/DMU) has become essential for train operation monitoring and fault diagnosis. However, this system is increasingly vulnerable to various cyber-physical threats, necessitating more intelligent and adaptive security protection mechanisms. This paper presents an intelligent security defense framework that integrates intrusion detection, risk scoring, and response mechanisms to enhance the security and responsiveness of the T2G communication system. First, feature selection is performed on the TON_IoT dataset to develop a Dream Optimization Algorithm (DOA)-optimized backpropagation neural network (DOA-BPNN) model for efficient anomaly detection. A Bayesian risk scoring module then quantifies detection outcomes and classifies risk levels, improving threat detection accuracy. Finally, a Q-learning-based reinforcement learning (RL) module dynamically selects optimal defense actions based on identified risk levels and attack patterns to mitigate system threats. Experimental results demonstrate improved performance in both multi-class and binary classification tasks compared to conventional methods. The implementation of the Bayesian risk scoring and decision-making modules leads to a 63.56% reduction in system risk scores, confirming the effectiveness and robustness of the proposed approach in an experimental environment.

## 1. Introduction

As intelligent rail transit systems evolve, the operational speed and density of high-speed trains are increasing rapidly. According to statistics from the International Union of Railways (UIC) [1], global rail passenger volume in 2023 grew by 3.5% compared to 2019, with the Asia-Pacific and Middle East regions accounting for over 78% of this increase. China’s high-speed rail system has expanded particularly quickly, reaching a total operating railway mileage of 162,000 km by the end of 2024, including 48,000 km of high-speed lines. Its annual passenger turnover has reached 922.6 billion passenger-kilometers, the highest in the world. Meanwhile, densely networked regions like Europe continue to play a key role in the global deployment of intelligent railway systems.

Against this backdrop, ensuring the operational safety and communication stability of Electric Multiple Unit/Diesel Multiple Unit (EMU/DMU) trains has become a critical challenge. The Train Control and Management System (TCMS), serving as the core control platform of modern trains, integrates key subsystems such as traction, braking, and doors through the Train Communication Network (TCN), while enabling real-time monitoring of operational states via maintenance terminals and human-machine interfaces (HMI). While TCMS has significantly improved operational efficiency through digitalization and networking, its external communication interfaces were not originally designed with cybersecurity in mind, leaving them vulnerable to attacks.

**Figure 1:**
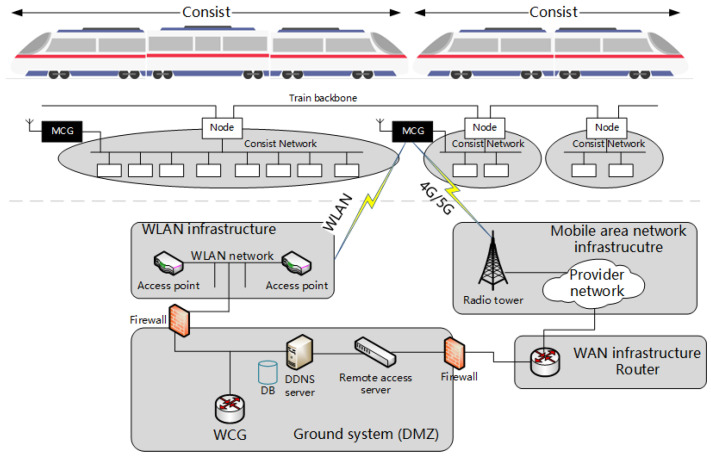
T2G communication system network structure.

Among these external interfaces, the Train-to-Ground (T2G) communication channel, in line with the reference architecture from IEC 61375-2-6, plays a central role in relaying real-time data from onboard equipment to ground data centers over 4G/5G or WLAN networks [2], as illustrated in Figure 1. On the train side, the Mobile Communication Gateway (MCG) handles routing, firewall management, and authentication for all outbound traffic, while the Wayside Communication Gateway (WCG) on the ground side manages secure entry from external networks and facilitates connections to back-end services. This channel is responsible for transmitting operational parameters, positional data, analysis data such as Electric Multiple Units Operation Analysis System (EOAS) data, diagnostic messages, and fault logs in real-time or in bulk. Although T2G data exchange typically employs encryption, its exposure to public or carrier-operated networks still leaves it vulnerable to various cyber-physical threats. Potential adversaries may intercept, alter, replay, or block these data flows, whether they be unauthorized individuals in public areas, malicious insiders, or compromised components in the supply chain. As a result, any compromise of the T2G communication system could lead to data leaks, communication failures, or even interruptions in core services.

To address the aforementioned challenges, this study proposes an intelligent security defense framework specifically designed for the T2G communication system to enhance the security and adaptive response capabilities of critical train communication links. The framework integrates a Dream Optimization Algorithm (DOA)-optimized backpropagation neural network (BP) for intrusion detection, a Bayesian risk scoring mechanism, and a reinforcement learning (RL)-based decision module, forming a closed-loop architecture encompassing detection, evaluation, and response. More specifically, the Industry 4.0/Internet of Things (IoT) and Industrial IoT (IIoT) dataset—TON_IoT—is adopted as the experimental foundation [3,4,5]. A hybrid feature selection strategy, combining logistic regression with forward sequential selection, is employed to extract a compact and highly discriminative feature subset. The DOA algorithm [6] is then used to optimize the initial weights and thresholds of the BP neural network [7], resulting in a high-performance intrusion detection model referred to as DOA-BPNN. On top of this, a Bayesian inference–based risk scoring module quantifies real-time threat levels based on detection outputs [8]. The resulting risk scores are fed into a reinforcement learning module, where a Q-learning algorithm dynamically generates optimal defense strategies according to the identified risk levels and attack patterns, thereby mitigating potential threats to the system [9].

The main contributions of this paper are summarized as follows:

- We analyze the security vulnerabilities of the T2G communication system to identify potential attack surfaces and abnormal behavior patterns.
- A hybrid feature selection strategy combining logistic regression and forward sequential selection is proposed to obtain a compact and relevant feature subset, thereby improving model efficiency and generalization.
- An intrusion detection model based on DOA-BPNN is developed. The DOA enhances the initialization of network weights, accelerates convergence, and reduces the risk of local minima. Simulation results show that the proposed method achieves improved detection accuracy and training stability compared with the standard BP model.
- A Bayesian risk scoring mechanism is introduced to quantify threat levels based on detection results. It incorporates detection confidence, anomaly intensity, and contextual adjustment to generate standardized real-time risk scores, enhancing threat perception and supporting dynamic defense decisions.
- A reinforcement learning–based response module is incorporated, using Q-learning to adaptively select optimal defense actions under varying risk levels and attack types. This enables closed-loop, feedback-driven responses, providing intelligent and scalable protection for the T2G communication system.
- To comprehensively evaluate the proposed framework, comparative experiments are conducted with other optimization algorithms in both binary and multi-class classification tasks. The framework’s effectiveness is also evaluated in scenarios with and without active response mechanisms.

The remainder of this paper is organized as follows. Section 2 reviews recent studies on train control system security and intrusion detection. Section 3 analyzes major threat models and attack classifications targeting the T2G communication system. Section 4 presents the proposed framework, detailing the dataset, feature selection method, and the detection–evaluation–response pipeline. Section 5 provides experimental results and analysis. Section 6 concludes the paper and outlines directions for future work.

## 2. Related Work

From the perspective of train operation, data transmission between onboard and ground systems in EMU/DMU can essentially be regarded as a wireless communication process. To date, most research on intrusion detection in railway wireless communication has focused on communication-based train control (CBTC) systems. Gao et al. [10] introduced a two-layer T2G intrusion detection; the first layer employs machine learning (RF/GBDT) on IEEE 802.11 traffic to spot attacks and the second layer uses a state observer to monitor key physical variables like movement authority. This approach minimizes false alarms while swiftly detecting threats that jeopardize train safety. A method was proposed that combines network status and device condition to identify anomalous traffic, with the added capability of distinguishing cyberattacks from random system faults [11], and their approach achieved a true positive rate (TPR) of 97.64%. He Lu et al. [12] developed a transfer learning–based intrusion detection system for CBTC, utilizing an optimized 1D convolutional neural network and long short-term memory network to automatically capture spatial and temporal features in network data. Simulated annealing was used for hyperparameter tuning, achieving an attack detection accuracy of up to 99.32%. Binyu Yin et al. [13] proposed a hybrid intrusion detection framework that integrates an improved stacking ensemble algorithm with a false-positive suppression strategy. By optimizing base classifier probabilities and applying Bayesian optimization for hyperparameter tuning, the method demonstrated excellent performance on both the KDD99 and CBTC simulation datasets. With the growing deployment of 5G, Wei Li et al. [14] investigated 5G-enabled intrusion detection strategies for CBTC. Their experiments showed that, whether using decision trees or convolutional neural networks, classification performance in both binary and multi-class tasks improved significantly after feature selection—highlighting the importance of proper data preprocessing and feature engineering for intrusion detection system (IDS) performance. Xiangyu Kong et al. [15] introduced a self-generated encoding technique that uses train message timestamps to dynamically generate encryption sequences. This method was designed to detect stealthy false data injection (FDI) attacks in the Train–Ground Communication System (TGCS), enabling the effective detection of anomalous activity. Amin Fakhereldine et al. [16] further extended intrusion detection research to TCMS by analyzing actual train operational data. They defined critical context features influencing intrusion detection within CBTC communication, including physical, network, environmental, and operational contexts. A variety of algorithms were evaluated, including Naive Bayes, logistic regression, k-nearest neighbors, random forest, XGBoost, isolation forest, local outlier factor, and elliptical envelope, all confirming the importance of context-aware information in train-related intrusion detection.

In recent years, researchers have employed a wide range of techniques for intrusion detection, including signature-based, anomaly-based, and behavior-based approaches, particularly for identifying botnet activity [17]. With the advancement of machine learning, IDS have increasingly incorporated machine learning algorithms to overcome the limitations of traditional detection methods [18]. Jiang H. et al. [19] proposed a network intrusion detection model based on Particle Swarm Optimization (PSO) and XGBoost, where PSO is used to automatically optimize the hyperparameters of the XGBoost classifier. The model was evaluated on the NSL-KDD dataset and demonstrated superior performance in identifying rare attack types such as User-to-Root (U2R) and Remote-to-Local (R2L), outperforming standard XGBoost, Bagging, and random forest classifiers in overall accuracy. Hsu et al. [20] developed a hybrid deep learning model that first employs convolutional neural network (CNN) layers to extract key features from the input data and then uses long short-term memory (LSTM) layers to capture temporal dependencies. Experimental results on the NSL-KDD dataset showed significant improvements in the detection accuracy of abnormal network activities, particularly in recognizing complex attack patterns.

Lu Lv et al. [21] introduced an intrusion detection method based on a Hybrid Kernel Extreme Learning Machine (HKELM), which combines radial basis function and polynomial kernels. Model parameters are optimized using a hybrid strategy that integrates Differential Evolution (DE) and Gravitational Search Algorithm (GSA), and feature reduction is performed via Kernel Principal Component Analysis (KPCA). The proposed method achieved excellent detection performance and computational efficiency across the KDD99, UNSW-NB15, and Tennessee Eastman (TE) industrial process datasets. Liu Tao et al. [22] proposed an intrusion detection model that combines an entropy-based Particle Swarm Optimization (EPSO) algorithm with a backpropagation neural network (BPNN). By dynamically adjusting the inertia weight of the PSO, the algorithm improved global search capability and significantly enhanced the optimization of weights and thresholds in the BP network. The EPSO-BPNN model achieved a detection accuracy of 92.90% on the KDD Cup 99 dataset, outperforming traditional BP, Support Vector Machine (SVM), and standard PSO-BPNN approaches.

Intrusion detection is commonly based on network traffic analysis, which requires processing features such as statistical attributes, correlation, behavioral patterns, payload content, and port information to identify suspicious activities. However, such data processing imposes a considerable computational burden on onboard train devices, which are typically resource-constrained in terms of energy and processing capacity [23]. Behavior-based detection approaches aim to discover potential threats—such as botnets—by identifying deviations from historical communication patterns. These methods often rely on a baseline of normal behavior, which can make it difficult to detect advanced persistent threats employing stealthy strategies [24]. Therefore, lightweight, efficient, and deployable IDS architectures are essential for train-mounted embedded systems [25]. To address this, a lightweight IDS framework was proposed, which utilizes a stochastic gradient descent classifier (SGDC) in combination with four ridge regression–based feature selection algorithms. Their system extracts the most effective feature subsets from large-scale datasets and achieves high detection performance while significantly reducing both training and testing time. The experimental results of three benchmark datasets—KDD-CUP-1999, BotI-oT-2018, and N-BaIoT-2021—demonstrate its effectiveness in constrained environments [26].

Following this approach, this study proposes a BPNN-based intrusion detection system optimized using the DOA algorithm. A Bayesian risk scoring mechanism is further incorporated to assess the threat level of each detected instance, and the results are fed into a reinforcement learning–based decision module. This closed-loop architecture allows for adaptive and optimal defense decisions for train operations, while minimizing security risks under limited computational resources.

## 3. T2G Communication Security, Vulnerabilities, and Threats

With the widespread adoption of wireless communication technologies in high-speed railway systems, the T2G communication system has become a vital component for real-time data exchange and fault monitoring. Cyberattacks targeting the T2G system can disrupt train operations, endanger passenger safety, cause property damage, and result in significant economic losses. Therefore, understanding the types of attacks that threaten the T2G system and assessing their potential impacts are essential for designing effective, intelligent defense strategies. To establish a practical foundation for threat modeling, the following five assumptions are made:

- The attacker possesses in-depth knowledge of the T2G system and may be capable of physically following the train;
- The attacker can impersonate legitimate communication entities or replay previously captured T2G data packets;
- The attacker is able to access communication devices and modify or relay data packets at different times and locations;
- The attacker may exploit radio equipment deployed along the railway to eavesdrop on, forge, or jam T2G communications;
- The attacker cannot physically dismantle, damage, or illegally relocate T2G devices without being detected.

### 3.1. Threats Overview

As a core component for status monitoring and data transmission in high-speed rail systems, the T2G communication system is particularly vulnerable to cyber threats due to its open architecture, mobile nature, and reliance on real-time wireless links. Based on the attacker model defined above, the major categories of threats faced by the T2G system can be summarized as follows:

1. Eavesdropping and Traffic Analysis: T2G communication often relies on public networks (e.g., 4G/5G), making it susceptible to passive attacks. Adversaries can deploy eavesdropping equipment along the railway to intercept and analyze transmitted packets. Even when encryption is applied, traffic analysis techniques (e.g., observing packet size, frequency, and timing) may reveal sensitive information such as train operating patterns, schedules, or equipment status.
2. Message Tampering and Forgery: Attackers may conduct man-in-the-middle (MitM) attacks by hijacking onboard communication channels or accessing network points at stations or along the track. This allows them to inject falsified operational parameters, fault reports, or location data, which could mislead the ground control system.
3. Replay Attack: Due to the periodic and predictable nature of T2G data transmissions, attackers can record legitimate packets and replay them at a later time to generate misleading operational states, potentially misleading decision-making on the ground side.
4. Denial-of-Service (DoS) Attacks: Attackers may disrupt T2G services by launching network-layer or physical-layer DoS attacks. These include flooding the channel with excessive traffic, exhausting system resources, or using high-power jamming signals. In busy station environments, such attacks could lead to widespread train delays and service disorganization.
5. Unauthorized Access and Device Compromise: Key T2G hardware components—such as Mobile Communication Gateways (MCGs), data acquisition units, and transmission controllers—may be compromised through supply chain vulnerabilities, misconfigurations, or leaked credentials. Without physically damaging the device, attackers could implant malicious code, enabling persistent access, data leakage, or remote shutdown of train communication functions.
6. Data Integrity Attacks: T2G data is often uploaded in batches for trajectory reconstruction, fault diagnostics, and performance analysis. Attackers could corrupt historical data or inject false supplementary records, undermining the consistency of datasets and impairing predictive maintenance or safety evaluations.
7. Routing and Protocol Exploits: In multi-train or edge-computing environments, T2G communication may leverage Software-Defined Networking (SDN), Mobile Edge Computing (MEC), or dynamic routing protocols. Adversaries may exploit protocol vulnerabilities to perform attacks such as route spoofing or cache poisoning, manipulating data paths or delaying service responses.
8. Mobile Network-Specific Threats: When relying on public 4G/5G infrastructures, T2G systems are also exposed to mobile-specific threats such as rogue base stations, DNS spoofing, and signaling storms. These techniques can intercept sessions, downgrade encryption, or force channel switching, potentially enabling more sophisticated or coordinated attacks.

### 3.2. Multi-Stage Advanced Attacks

During the operation of EMU/DMU trains, the T2G system continuously uploads high-frequency data—including train status, location, fault parameters, and energy consumption—to ground control centers. Upon entering a depot state (such as a maintenance facility or parking area), the system transitions to batch data synchronization, device self-diagnostics, and firmware maintenance mode. Unlike traditional single-point attacks, real-world adversaries often orchestrate multi-stage or multi-vector attacks by combining several lightweight techniques to progressively infiltrate the system while remaining below the fault-tolerance threshold. These advanced attack strategies are typically characterized by high stealth and persistence, posing significant challenges for both detection and defense. Below are several representative combined attack scenarios specifically targeting T2G communication:

1. Forgery-Jamming Combination During Online Operation: While the train is in active service, attackers may monitor T2G transmissions to infer data formats and forge onboard status messages with structurally identical characteristics. Short-term jamming—achieved via high-frequency interference or intentional data congestion—is then introduced during legitimate message transmission windows, preventing the ground system from receiving authentic data. As a result, the forged message may be erroneously accepted as the “current train status”. This attack vector can be exploited to simulate faults, mislead train scheduling systems, or conceal actual anomalies.
2. Low-Frequency Injection with Replay: During normal operation, attackers intermittently inject low-frequency anomalies—such as borderline temperature, voltage, or current fluctuations—to mimic natural operational variations without triggering alarms. Once the train enters a depot for centralized data upload, historical “normal” messages may be replayed or combined with fabricated records, introducing logical inconsistencies in the dataset. This undermines predictive maintenance algorithms and health monitoring processes, potentially resulting in incorrect lifecycle estimations, false alarms, or unnecessary maintenance interventions.
3. Sequence Tampering and Timestamp Drift: T2G data packets transmitted during high-speed operation often contain critical temporal metadata such as timestamps and sequence numbers, which are essential for event reconstruction by ground-based systems. By subtly modifying timestamps (e.g., shifting by a few seconds) or scrambling the order of transmission, attackers can distort causal sequences (e.g., “braking → acceleration → fault alarm”), leading to misinterpretation of events and compromising situational awareness and decision-making on the ground.

### 3.3. Attack Classification

**Figure 2:**
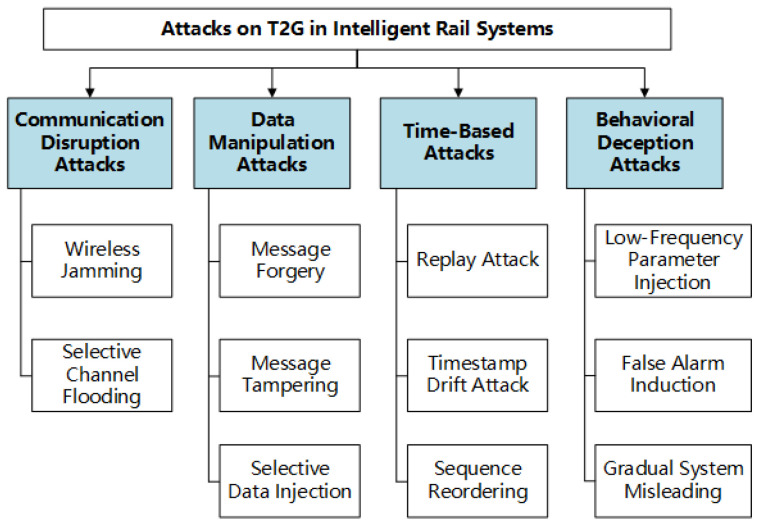
Attack classification of T2G.

To standardize the categorization of the aforementioned attack types, the primary threats faced by the T2G system can be classified into four major categories based on attack behaviors and targeted objectives: communication disruption, data manipulation, temporal control, and behavioral deception, as illustrated in Figure 2.

## 4. Methodology

This study aims to develop an intelligent security defense framework for T2G, which integrates intrusion detection, risk scoring, and response decision execution to enhance cybersecurity protection during train operations. This section presents the overall architecture of the proposed framework, the datasets used, experimental settings, feature selection techniques, and the detailed implementation of the model.

### 4.1. Proposed System Overview

**Figure 3:**
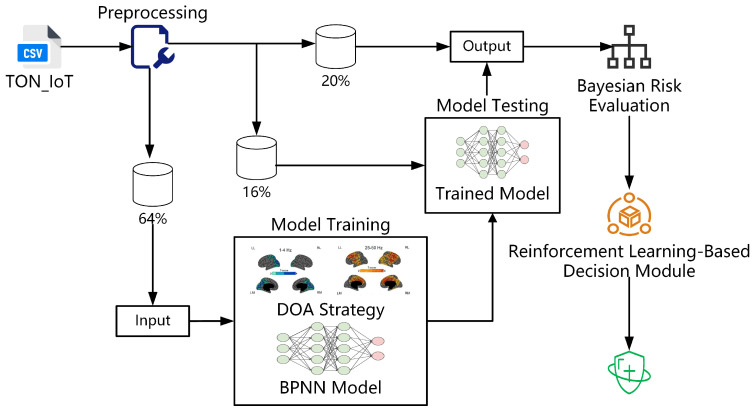
Workflow of DOA-BPNN and RL-based T2G protection system.

To effectively enhance the detection and response capabilities of T2G communication system against cyber intrusions, this study proposes an intelligent security defense framework that integrates intrusion detection, risk scoring, and decision execution. Based on the TON_IoT dataset, the framework incorporates a DOA-BP intrusion detection model, a Bayesian risk scoring module, and a reinforcement learning-based decision-making module to identify potential threats and dynamically generate response strategies for T2G systems. As illustrated in Figure 3, the framework consists of three main stages: data acquisition and preprocessing, intrusion detection, and risk evaluation with corresponding decision execution.

Stage One: The latest high-quality IoT security dataset, TON_IoT, is selected as the experimental dataset to simulate the communication behavior of transmission devices in the T2G environment of EMU/DMU systems. This stage involves data import, preprocessing, partitioning, and feature construction.

Stage Two: This study introduces DOA to initialize parameters and optimize the structure of the classical BPNN, resulting in an efficient and stable intrusion detection model that outputs classification labels.

Stage Three: Since intrusion detection results alone cannot directly guide system behavior, further evaluation of the potential risk caused by the intrusion is required. Risk levels are quantified based on attack types, posterior probabilities, and contextual state features. The system then activates the Reinforcement Learning-Based Decision Module, which dynamically generates optimal response actions (such as blocking, isolation, or logging) according to the evaluation results. This module iteratively improves its policy to achieve adaptive feedback defense.

### 4.2. Dataset Description

Selecting an appropriate dataset is essential for evaluating the performance of IDS. Several publicly available datasets have been widely used in IDS research, such as TON_IoT, KDD Cup99 [27], and UNSW-NB15 [28,29]. The KDD Cup99 dataset was initially introduced at the Fifth International Conference on Knowledge Discovery and Data Mining (KDD-99) in 1998 as part of the Third International Knowledge Discovery and Data Mining Tools Competition. As one of the earliest benchmark datasets for intrusion detection, it played a foundational role in early studies. However, with the evolution of network threats and system complexity, this dataset is now limited by its outdated feature distributions, class imbalance, and lack of diversity in attack types, making it less suitable for evaluating modern Network-Based Intrusion Detection System (NIDS) models [30]. The UNSW-NB15 dataset was created using the IXIA PerfectStorm tool in the Cyber Range Lab of UNSW Canberra. It blends real-world background traffic with synthesized modern attack behaviors, offering a comprehensive representation of contemporary internet communication.

Building on the previous work, the TON_IoT dataset was collected in a realistic, large-scale test environment at the UNSW Canberra Cyber Institute’s IoT Lab in 2019. It was specifically designed for research into the security of IoT and IIoT systems. This dataset integrates heterogeneous data from multiple system platforms, including operating system logs, telemetry from sensors, network traffic records, and behavioral sequences, comprehensively capturing both normal and malicious patterns found in typical industrial intelligent environments [31,32]. Given its rich data structure and practical relevance—closely resembling the complex communication interactions between devices and platforms in the T2G environment of EMU/DMU—it features high dimensionality, strong temporal characteristics, diverse attack types, and controllable sample distribution. In this study, the Processed Network subset of TON_IoT was selected as the primary experimental dataset to support the construction and validation of the proposed intelligent intrusion detection and response framework. In this work, we utilize a 10% subset of the TON_IoT dataset to reduce computational overhead while preserving sufficient data representativeness. This subset was selected using stratified random sampling to maintain the original class distribution proportions. Table 1 shows the data structure of the TON_IoT dataset we used.

### 4.3. Dataset Pre-Processing

To improve dataset suitability and experimental reliability, the original data were processed as follows:

1. Data Cleaning: Duplicate records and samples with more than 75% missing values were removed to reduce noise and improve data quality.
2. Dataset Splitting: The dataset was first split into two parts, with 80% allocated to the DOA-BP intrusion detection module and the remaining 20% reserved for the Bayesian risk scoring and reinforcement learning–based response modules. Within the 80% assigned to intrusion detection, an additional 80:20 split was applied to create the training and test sets. This two-stage partitioning yields a final distribution of 64% for training, 16% for testing, and 20% for evaluation of downstream components. This hierarchical strategy ensures data independence between modules, prevents information leakage, and supports unbiased performance evaluation. Stratified sampling was employed throughout to maintain consistent class distribution across all subsets.
3. Class Balancing: Stratified random sampling maintained representativeness. All samples of rare attack types (e.g., mitm, ransomware, injection) were retained. For normal traffic, a 20% sampling rate was applied, and for other common attacks, 10% was used to balance data volume and computational efficiency.
4. GAN-Based Augmentation: For attack types with very few samples (e.g., mitm, with only 1473 samples), a Generative Adversarial Network (GAN) was used to create additional synthetic data, enhancing generalization by matching the original distribution.
5. Feature Engineering and Encoding: Non-numeric fields were converted into numeric format, and formatted numeric fields were cleaned and standardized. Categorical features were label-encoded, and mappings were saved to ensure interpretability and traceability. Finally, all numeric features were normalized to the [0, 1] range using min–max scaling to standardize the input for model training.

### 4.4. Feature Selection

In large-scale, high-dimensional Industrial IoT datasets such as TON_IoT, feature selection plays a crucial role in improving the performance of intrusion detection systems. By eliminating redundant or irrelevant features and retaining those strongly correlated with the target variable, the model’s detection accuracy can be enhanced while significantly reducing training time and computational overhead. To address the “curse of dimensionality” associated with high-dimensional data, this study adopts a hybrid feature selection approach. First, the raw data undergo a coarse filtering stage, followed by a more refined selection process using logistic regression and Forward Feature Selection to identify the most representative feature subset [33]. Before finalizing this hybrid approach, we conducted a comparative evaluation of ten feature selection methods, including correlation analysis, feature importance ranking using random forest, logistic regression and Ridge Classifier, as well as forward selection and backward selection combined with these classifiers. Logistic regression with Forward Feature Selection consistently outperformed the alternatives in balancing detection accuracy and computational efficiency. This method iteratively evaluates the contribution of each new feature to classification performance, retaining only the minimal subset that achieves optimal accuracy.

The original TON_IoT dataset contains 44 features. After applying the proposed selection strategy, 20 features were identified as having strong correlations with the target class (i.e., normal vs. attack types), demonstrating high utility for anomaly detection tasks. This reduced feature subset was subsequently used for training and testing the DOA-BP model. The selected feature count is summarized in Table 2.

### 4.5. Dream Optimization Algorithm (DOA)

The Dream Optimization Algorithm (DOA) is a metaheuristic algorithm inspired by the process of human dreaming during REM sleep. It simulates three key cognitive behaviors: memory retention, partial forgetting with supplementation, and dream-sharing. The algorithm operates in two main phases—exploration and exploitation. The key steps in the DOA optimization process are as follows:

Step 1: Initialization. DOA begins by randomly generating an initial population within the predefined bounds of the search space. Each individual is initialized using the formula:

(1) $$X_{i}=X_{l}+rand\cdot \left(X_{u}-X_{l}\right),i=1,2,\ldots ,N$$

where $X_{l}$ and $X_{u}$ are the lower and upper bounds of each dimension and rand is a uniformly distributed random vector.

Step 2: Exploration Phase. During the early stage of the optimization (iteration $t<T_{d}$), the population is divided into five groups with different memory capacities. Individuals first reset their position to the best solution found in their group:

(2) $$X_{i}^{t+1}=X_{bestq}^{t}$$

Then, in selected dimensions $j\in K_{1},\ldots ,K_{k_{q}}$, partial forgetting and supplementation are performed using the cosine-guided strategy:

(3) $$x_{i,j}^{t+1}=x_{bestq,j}^{t}+\left(x_{l,j}+rand\times \left(x_{u,j}-x_{l,j}\right)\right)\times \frac{1}{2}\times \left(cos\left(\pi \times \frac{t+T_{max}-T_{d}}{T_{max}}\right)+1\right),j=K_{1},K_{2},\ldots ,K_{k_{q}}$$

Additionally, the dream-sharing mechanism allows individuals to acquire dimension-specific information from other members of the population:

(4) $$X_{i,j}^{t+1}=\left\{\begin{array}{cc} x_{m,j}^{t+1}, & m\leq i\\ x_{m,j}^{t}, & i<m\leq N \end{array}\right.$$

Step 3: Exploitation Phase. After $T_{d}$, all individuals follow the globally best solution. The memory retention is updated by:

(5) $$X_{i}^{t+1}=X_{best}^{t}$$

For the same selected dimensions, the forgetting and supplementation strategy is reused with a modified cosine schedule:

(6) $$x_{i,j}^{t+1}=x_{best,j}^{t}+\left(x_{l,j}+rand\times \left(x_{u,j}-x_{l,j}\right)\right)\times \frac{1}{2}\times \left(cos\left(\pi \times \frac{t}{T_{max}}\right)+1\right),j=K_{1},K_{2},\ldots ,K_{k_{q}}$$

Step 4: Fitness Evaluation and Iteration. At each iteration, the fitness of all individuals is evaluated. The algorithm iteratively updates the population using the above strategies until a predefined maximum number of iterations $T_{max}$ is reached.

### 4.6. DOA-BPNN Model

**Figure 4:**
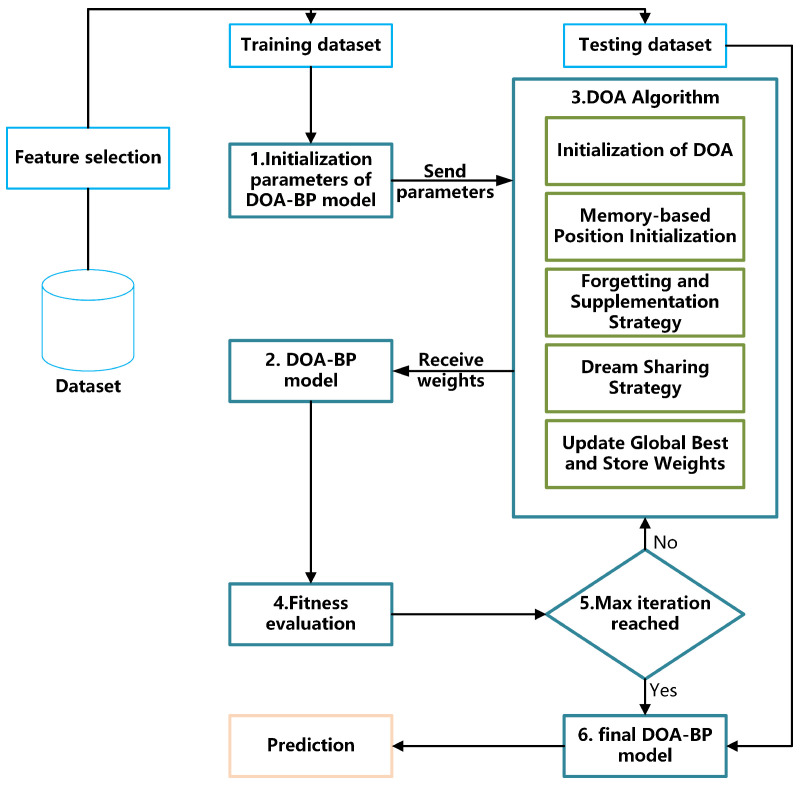
Workflow of the DOA-BPNN model.

This section presents the proposed DOA-BPNN model, in which the DOA algorithm is used to globally optimize the weights and biases of the BP neural network. Inspired by brain-like cognitive behavior, DOA improves the quality of weight initialization, thereby solving common limitations of traditional BP networks, such as vulnerability to local minima and slow convergence. The optimized weights and biases serve as the initial parameters for the network, which are further refined using the classical backpropagation algorithm to enhance convergence precision and robustness. The overall framework of the DOA-BPNN model is illustrated in Figure 4.

Step 1: DOA-BPNN Model Initialization. The model begins by defining the topology of the BP neural network, including the number of input dimensions (corresponding to the selected features), the number of neurons in the hidden layer, and the number of output classes. The initial weights and bias vectors of the neural network are encoded as parameters in the optimization space of the DOA algorithm.

Step 2: DOA Parameter Initialization. The required parameters for the DOA algorithm are set, including population size, dream dimensions, forgetting rate, and the number of iterations. All individuals in the population are randomly initialized, with each encoding a set of neural network weights and biases. The fitness evaluation criterion is defined directly using the classification accuracy, where higher accuracy reflects better fitness, aligning the optimization goal with maximizing detection performance.

Step 3: Iterative Weight Optimization and Fitness Evaluation. In each iteration, DOA performs memory recall, forgetting and reconstruction, and dream-sharing strategies to generate a new population of candidate solutions. Each individual represents a combination of weights and biases, which are applied to the BP neural network for forward propagation. The classification error is then computed as the fitness score and returned to the optimizer.

Step 4: DOA-Based Weight Update. The best solution is updated based on the fitness evaluations, and individual dimensions are adjusted. The optimization terminates when the maximum number of iterations is reached or if no improvement is observed over several consecutive generations.

Step 5: BP Network Training and Refinement. The optimal weights and biases obtained from DOA are used in the BP neural network as initialization. The model is then trained using the standard backpropagation algorithm on the training set to further refine the parameters and improve classification accuracy.

Step 6: Model Prediction and Output. Upon completion of training, the DOA-BPNN model is used to predict test data labels (attack types). The results serve as input to the subsequent risk scoring and response decision modules.

Specifically, in our DOA-BPNN, the parameter optimization problem is defined as a search space optimization problem:

(7) $$\min_{\omega \in \Omega }F\left(\omega \right)=1-A_{cc}\left(\hat{y},y\right)$$

where ω represents the parameters of BPNN, including weights matrices *W*, biases vectors *b*, $A_{cc}\left(\cdot ,\cdot \right)$ is the accuracy measurement function, and $\hat{y}$ is the output of BPNN when the input is *X*.

First, we initialize the BPNN parameters:

(8) $$X_{i}=W_{init}+\delta_{i},i=1,2,\ldots ,N_{pop}$$

where $W_{init}$ is the BPNN initialization parameter, $\delta_{i}∼U{\left(-1,1\right)}^{D}$ is a uniformly distributed random vector, and $N_{pop}$ is the population size.

Next, we apply the DOA algorithm to optimize BPNN. After initializing the total number of iterations $T_{max}$, with the exploration phase boundary defined as $T_{d}=0.8\cdot T_{max}$, and the probability of random forgetting u=0.6, the optimization proceeds in two phases: exploration and exploitation. During the exploration phase ($t<T_{d}$), varying levels of forgetting intensity are assigned to each population group, and parameters are updated according to the following steps.

Step1: Find the optimal solution within the group as the guiding solution:

(9) $$X_{q,best}^{t}=\arg\max_{X_{i}^{t}\in G_{q}}F\left(X_{i}^{t}\right)$$

where $F(X_{i}^{t})$ is the fitness value of individual $X_{i}$, representing the validation accuracy. The population of dreams is divided into 5 groups, following the original DOA formulation, to balance exploration diversity and convergence stability.

Step 2: Calculate the number of forgetting dimension based on the current iteration and group number:

(10) $$k_{q}\left(t\right)=\left[1+\left(D-1\right)\cdot \left(1-\frac{t}{T_{d}}\right)\cdot \left(\frac{5-q}{5}\right)\right]$$

where *D* is the total number of parameter dimensions, *t* is the current iteration, $T_{d}$ is the total number of iteration in the exploration phase, and q∈{0,1,2,3,4} represents the group index.

Step 3: For each individual $X_{i}^{t}$ in $G_{q}$, apply the memory strategy to preserve the optimal structure in the group:

(11) $$X_{i}^{t+1}=X_{q,best}^{t}$$

Step 4: Randomly select $k_{q}\left(t\right)$ dimensions to form the forgetting index set $\Omega_{forget}$, then perform random forgetting with probability *u* and dream sharing with probability 1−u. Only the dimensions in $\Omega_{forget}$ are modified, while other dimensions remain unchanged:

(12) $$X_{i}^{t+1}\left(j\right)=\left\{\begin{array}{cc} U(lb_{j},ub_{j}), & \mathrm{if}\;j\in \Omega_{forget}\\ X_{i}^{t+1}\left(j\right), & \mathrm{else} \end{array}\right.$$

(13) $$X_{i}^{t+1}\left(j\right)=\left\{\begin{array}{cc} X_{r}^{t}\left(j\right), & \mathrm{if}\;j\in \Omega_{forget},r\neq i\\ X_{i}^{t+1}\left(j\right), & \mathrm{else} \end{array}\right.$$

where *r* is the index of another randomly selected individual from population.

Step 5: Combine the memory part and forgetting part to update the individual’s position:

(14) $$X_{i}^{t+1}=clip\left(X_{i}^{t+1},lb,ub\right)$$

When the number of iterations reaches the threshold value $T_{d}$, the DOA-BPNN enters the exploitation phase, where the focus shifts to fine-tuning the global optimal solution. The steps are as follows:

Step 6: Determine the current global optimal solution:

(15) $$X_{best}^{t}=\arg\max_{X_{i}\in X_{1},X_{2},\ldots ,X_{N_{p}op}}F\left(X_{i}\right)$$

Step 7: As iterations continue, gradually decrease the forgetting dimension number to enhance local search for the current optimal solution:

(16) $$k_{r}\left(t\right)=\left[1+\left(k_{max}-1\right)\cdot \left(1-\frac{t-T_{d}}{T_{max}-T_{d}}\right)\right]$$

where $k_{max}=\left\lfloor k\cdot D\right\rfloor$ represents the maximum number of forgetting dimensions. Considering the typical parameter scale of BPNN, we set k=2%, which provides a balanced exploration–exploitation trade-off.

Step 8: Apply memory strategy, with all representatives based on the global optimal solution:

(17) $$X_{i}^{t+1}=X_{best}^{t}$$

Step 9: Randomly select $k_{r}\left(t\right)$ dimensions to form a forgetting index set $\Omega_{forget}$, and apply the corresponding forgetting strategy. Only the dimensions in $\Omega_{forget}$ are modified, while other dimensions remain unchanged:

(18) $$X_{i}^{t+1}\left(j\right)=\left\{\begin{array}{cc} X_{best}^{t}\left(j\right), & \mathrm{if}\;j\in \Omega_{forget}\\ X_{i}^{t+1}\left(j\right), & \mathrm{else} \end{array}\right.$$

Step 10: Similar to the exploration phase, the individual’s position is then processed using the clipping mechanism described in Equation (14).

During the above parameter updates, boundary constraint clips are always used to ensure that the update solution remains within the valid search space.

A key innovation of our DOA-BP implementation is the “optimize-while-training” mechanism, where each dream (solution) undergoes a brief training phase before fitness evaluation:   

(19) $$W_{i}^{t+1}=\text{BP-Train}\left(W_{i}^{t},D_{train},lr=0.001\right)$$

where BP-Train represents one epoch of backpropagation training using Adam optimizer with learning rate η=0.001. This mechanism allows each dream to undergo local improvement through gradient descent, before being evaluated and subjected to the DOA operations, creating a synergy between gradient-based and gradient-free optimization.

The workflow of the proposed DOA-BPNN scheme is depicted in Algorithm 1.

**Algorithm 1** DOA Optimization for BPNN Parameters
  1:**Input**: Initial BPNN parameters $W_{init}$, Training dataset $D_{train}$, DOA parameters: {$N_{pop}$, $T_{max}$, $T_{d}$, *u*, lb,ub}  2:**Output**: Optimized BPNN parameters $W_{best}$  3:Compute dimension *D* = number of parameters in BPNN  4:

$k_{max}\leftarrow \left\lfloor k\cdot D\right\rfloor$

  5:for t←1 to $T_{max}$ do  6:      if $t<T_{d}$ (Exploration Phase) then  7:            Divide population into 5 groups:$G_{0},G_{1},\ldots ,G_{4}$  8:            for q←0 to 4 do  9:                  $X_{q,best}^{t}\leftarrow \arg\max_{X_{i}^{t}\in G_{q}}F\left(X_{i}^{t}\right)$10:                  $k_{q}\left(t\right)\leftarrow \left[1+\left(D-1\right)\cdot \left(1-\frac{t}{T_{d}}\right)\cdot \left(\frac{5-q}{5}\right)\right]$11:                  for each $X_{i}$ in $G_{q}$ do12:                        $X_{i}^{t+1}\leftarrow X_{q,best}$13:                        $\Omega_{forget}\leftarrow$ Randomly select $k_{q}\left(t\right)$ dimensions14:                        if rand()<u then15:                              Random forgetting16:                        else17:                              Dream sharing18:                        end if19:                        $X_{i}^{t+1}\leftarrow clip\left(X_{i}^{t+1},lb,ub\right)$20:                        update $F(X_{i}^{t+1})$21:                  end for22:            end for23:      else (Exploitation Phase)24:      $X_{best}^{t}\leftarrow \arg\max_{X_{i}\in \left\{X_{1},X_{2},\ldots ,X_{N_{pop}}\right\}}F\left(X_{i}\right)$25:            $k_{r}\left(t\right)\leftarrow \left[1+\left(k_{max}-1\right)\cdot \left(1-\frac{t-T_{d}}{T_{max}-T_{d}}\right)\right]$26:            for i←1 to $N_{pop}$ do27:                  $X_{i}^{t+1}=X_{best}^{t}$28:                  $\Omega_{forget}\leftarrow$ Randomly select $k_{r}\left(t\right)$ dimensions29:                  Apply forgetting strategy30:                  $X_{i}^{t+1}$ = $clip(X_{i}^{t+1},lb,ub)$31:                  update $F_{i}^{t+1}$32:            end for33:      end if34:      if $F_{i}^{t+1}>F_{best}$ then35:            $F_{best}\leftarrow F\left(X_{i}^{t+1}\right)$36:            $W_{best}\leftarrow X_{i}^{t+1}$37:      end if38:end for39:Return $W_{best}$


### 4.7. Bayesian Risk Scoring

In the context of high-speed railway operations, ensuring the security of train control systems is critical. Although IDS can effectively identify potential malicious activities, their outputs typically lack quantitative assessment of attack consequences, limiting their usefulness for informing system-level response strategies. To address this, we propose a Bayesian risk scoring method that follows the fundamental principle of quantitative risk assessment—representing overall risk as the product of relevant risk factors. According to George and Renjith [34], security risk evaluations commonly adopt multi-factor multiplicative models such as R=L×A×V×C, where each factor represents risk (*R*), likelihood of an attack (*L*), attractiveness of an asset (*A*), vulnerability in the facility (*V*), and the potential consequence (*C*) of a successful attack, respectively.

Inspired by this principle, our Bayesian risk scoring model integrates multiple critical factors derived from intrusion detection results in the T2G context. Specifically, it combines the impact score ($I_{i}$), posterior probability of the attack type($P_{i}$), and anomaly score ($A_{i}$), along with a context adjustment coefficient ($C_{i}$) that enhances sensitivity to high-confidence threats. This yields a standardized risk score and corresponding risk level label for each sample, calculated as

(20) $$Risk_{i}=I_{i}\times P_{i}\times A_{i}\times C_{i}\times 100$$

where $Risk_{i}$ denotes the risk score of the *i*-th sample, normalized to the range [0, 100]; $I_{i}$ reflects the potential impact of the detected attack on the T2G system; $P_{i}$ is the posterior probability of the *i*-th sample belonging to the predicted attack class, i.e., the confidence output by the DOA-BPNN; $A_{i}$ quantifies behavioral deviation from normal patterns; and $C_{i}$ is a context adjustment coefficient used to amplify risks under high-risk conditions. The thresholds for $A_{i}$ and $C_{i}$ are set to 0.8 and 1.2, respectively, guided by the Pareto Principle and expert knowledge [35]. When the anomaly score exceeds 0.8, the sample is highly likely to represent a real threat that warrants special attention. The context adjustment coefficient $C_{i}$ increases the risk weighting by 20% in such cases, aiming to balance between avoiding excessive false alarms and ensuring that high-risk threats receive sufficient priority. This design allows the system to allocate resources more effectively toward detections most likely to represent genuine security risks.

For samples predicted as normal, the risk score is simplified as

(21) $$Risk_{i}=A_{i}\times 100$$

Finally, the resulting risk scores are categorized into four predefined risk levels, as shown in the corresponding Table 3.

### 4.8. Reinforcement Learning-Based Response Decision Model

Following the risk score computation in the previous section, this section presents reinforcement learning (RL) techniques to construct a Q-learning-based response decision module to optimize the system’s adaptive response strategies based on different risk levels and attack types specific to T2G communication system, as described in Section 3. The RL environment is represented as a Markov Decision Process (MDP), in which the state space consists of two main factors: risk score and attack type confidence. To reduce training complexity, the risk score is discretized into 20 levels, and 10 types of attacks are treated as independent categorical dimensions, creating S=20×10=200 finite states. The action space consists of five types of defensive responses targeted at the onboard T2G devices, as listed in Table 4.

A further explanation of these decisions is provided as follows:

1. Monitoring and Logging: This action records and monitors train status and communication activities to detect threats such as eavesdropping and traffic analysis or unauthorized access, which correspond to backdoor attacks in the TON_IoT dataset. By tracking data flows between the train and ground systems, it helps identify unusual communication patterns or unauthorized access attempts in real-time.
2. Limiting Train Communication: In response to DoS/DDoS or communication jamming attacks, which are related to DDoS or flooding attacks in the TON_IoT dataset, this action limits the transmission rate of non-critical data, such as diagnostic messages, ensuring that crucial T2G control data, like operational commands or fault information, are prioritized and protected from overload.
3. Train Deceleration: If a MitM or message tampering attack is detected, which could correspond to MitM or injection attacks in the TON_IoT dataset, this action automatically initiates deceleration to ensure the train operates safely by reducing speed. This allows time to verify data integrity and control the situation before a critical failure occurs.
4. Emergency Braking: In cases of replay attacks or data integrity attacks, similarly to replay or ransomware attacks in the TON_IoT dataset, this action triggers emergency braking to rapidly stop the train, ensuring that any compromised operational data, such as false fault reports or location data, do not lead to unsafe situations, thereby protecting passengers and infrastructure.
5. System Reset: If a device compromise or routing exploit (similar to backdoor or command injection attacks in the TON_IoT dataset) is detected, this action performs a secure reboot or restoration of the affected T2G devices, such as MCG, to ensure the systems are cleared of any malicious modifications and return to a secure state, allowing communication to resume safely.

The training process uses a classical ε-greedy policy to dynamically balance exploration and exploitation. The specific steps are as follows:

Stage 1: Initialize the Q-table as a zero matrix.

Stage 2: Select a state randomly and observe the current state $s_{t}$; choose an action $a_{t}$ based on the ε-greedy probability.

Stage 3: Execute the selected action and evaluate its impact on system risk, receiving the corresponding reward $r_{t}$ and transition to a new state $s_{t+1}$.

Stage 4: Update the Q-table using the Q-learning update rule:

(22) $$Q\left(s_{t},a_{t}\right)\leftarrow Q\left(s_{t},a_{t}\right)+\alpha \left[r_{t}+\gamma \cdot \max_{a}Q\left(s_{t+1},a\right)-Q\left(s_{t},a_{t}\right)\right]$$

where α is the learning rate and γ is the discount factor. Once training is complete, the optimal policy is extracted by selecting actions with maximum Q-values for each state: $\pi^{*}\left(s\right)=\arg\max_{a}Q\left(s,a\right)$.

To balance risk mitigation with resource efficiency, the reward function is designed as

(23) $$r_{t}=R_{r}-\frac{risk}{100}\times C_{a}\times 50-P_{p}$$

where $R_{r}=risk\times E_{a}$ denotes the amount of risk reduction, $E_{a}$ represents the effectiveness of the chosen action, $C_{a}$ is the relative resource cost of the defense action, and $p_{p}$ is a penalty term for inappropriate strategies. If an overreaction or underreaction is detected (e.g., executing a system reset for a low-risk sample), then $P_{p}$ = 10; otherwise, $P_{p}$ = 0.

## 5. Experiments and Analysis of Results

This section presents the experimental setup and comprehensive analysis of the results achieved with the proposed model. Section 5.1 outlines the experimental environment and implementation details. Section 5.2 introduces the evaluation metrics used to assess model performance. Section 5.3 discusses the detection results of the proposed DOA-BPNN model, including its effectiveness in identifying various types of attacks. Furthermore, a comparative analysis is performed against several baseline techniques to demonstrate the model’s advantages.

### 5.1. Experimental Setup

All experiments were conducted on a workstation featuring an 11th-generation Intel Core i7 processor (2.30 GHz), 32 GB of RAM, and an NVIDIA GeForce RTX 3060 GPU, running on the Windows 10 operating system. The software environment was built using Python 3.10 within the Jupyter Notebook interface. The implementation relies on a combination of widely-used libraries including NumPy, PyTorch, Pandas, and Scikit-learn for data handling, model training, and evaluation. For fairness in comparison, the experimental parameters for all models, including the DOA-BP model and the other metaheuristic optimization algorithms, were set to be identical. These settings are summarized in Table 5, and are used consistently throughout the experiments to ensure a fair evaluation of the methods.

### 5.2. Evaluation Metrics

To evaluate the classification performance of the proposed DOA-BPNN model, we employ four widely used metrics: Accuracy, Precision, Recall, and F1-score. Accuracy measures the overall correctness of predictions [36], while Precision and Recall reflect the model’s ability to correctly identify positive instances and minimize false-negatives, respectively. F1-score provides a balanced measure by combining Precision and Recall. For the multi-class intrusion detection task, we report macro-averaged results across all classes to ensure fair evaluation, regardless of class imbalance.

### 5.3. Performance of DOA-BPNN Model

**Figure 5:**
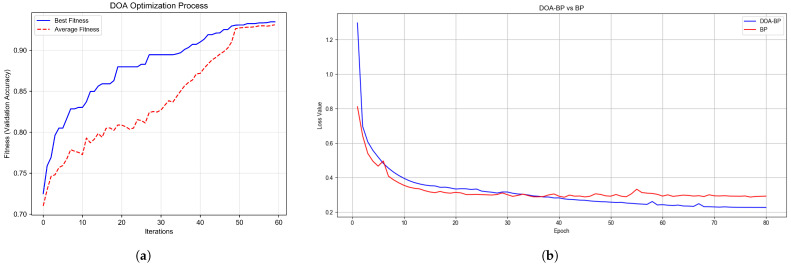
Performance of DOA-BPNN training process: (**a**) accuracy evolution during DOA-based parameter optimization; (**b**) loss convergence trend in the training of BP neural network initialized with DOA-optimized weights.

To evaluate the effectiveness of the proposed model in intrusion detection tasks, experiments were conducted under both multi-class and binary classification settings using consistent parameter configurations. As shown in Figure 5a, the DOA optimization process shows a steadily increasing trend in both best fitness and average fitness, with best validation accuracy exceeding 90% after 37 iterations and ultimately reaching 93.47% by iteration 60. This demonstrates the optimizers’s ability to progressively improve parameter quality over time. Figure 5b compares the training processes of the BP neural network with and without DOA-optimized initialization. The DOA-BP model achieves faster convergence and a lower final loss value compared to the standard BP network, highlighting that incorporating DOA optimization into the BP framework enhances overall training effectiveness and detection performance.

**Figure 6:**
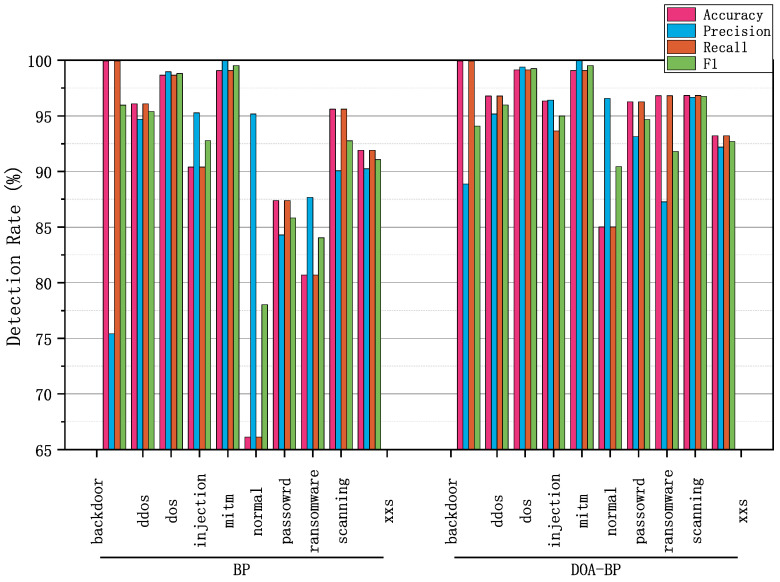
Comparison of the DOA-BPNN with BPNN in multi-class detection.

As shown in Figure 6, the proposed DOA-BP model outperforms the conventional BP neural network across almost all attack classes in the multi-class detection task. It achieves higher accuracy, precision, recall, and F1-scores in most classes, with only a slight F1-score decrease observed in the Backdoor class, from 95.97% to 94.09%, despite maintaining the same high accuracy of 99.95%. Notably, in challenging classes such as Normal and Password, DOA-BP delivers significant improvements. For Normal, accuracy and recall increase from 66.12% to 85.01%, and F1-score rises from 78.03% to 90.43%, indicating that when DOA-BPNN is applied to intrusion detection in T2G communication systems, it can more accurately recognize normal communication behaviors, thereby reducing the impact of false alarms that could trigger unnecessary safety measures. For Password, accuracy improves from 87.39% to 96.25%, and F1-score from 85.82% to 94.67%, showing the model’s effectiveness in detecting attacks targeting access control vulnerabilities in T2G communication systems, which are a critical security concern. In the Ransomware class, DOA-BP also shows strong gains, with accuracy increasing from 80.69% to 96.82% and F1-score from 84.04% to 91.80%, indicating a notable improvement in detecting data integrity threats that are crucial in railway operations. Despite these improvements, the Normal class remains the weakest point for both models, as its accuracy and recall, while improved, are still the lowest among all classes. This suggests that distinguishing benign traffic from actual threats remains challenging and warrants further investigation. Overall, these results highlight the consistent performance enhancements achieved by DOA-BP, while also pointing to key areas for future optimization, such as further refining detection for benign traffic and attacks targeting access control vulnerabilities in T2G communication systems.

**Figure 7:**
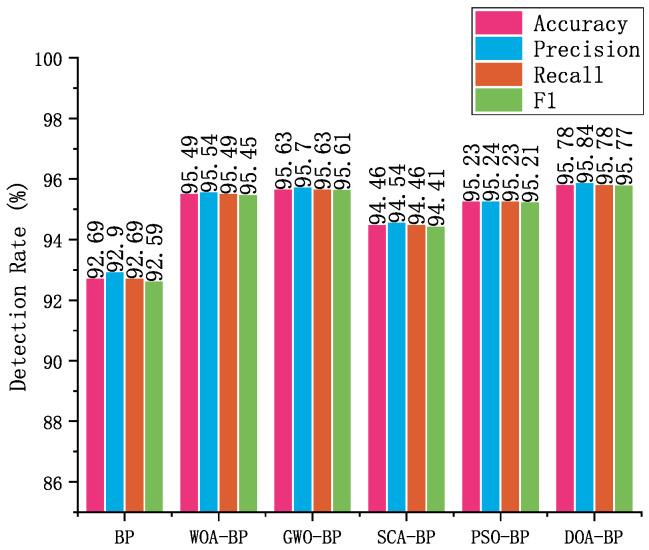
Comparison of the DOA-BPNN with other optimization algorithm in binary classification.

To further assess the effectiveness of the proposed DOA-BP model, it was compared against BP neural networks optimized by four commonly used metaheuristic algorithms: WOA [37], GWO [38], SCA [39], and PSO [40]. Figure 7 shows that all optimization methods improve upon the baseline BP network, with GWO-BP and DOA-BP exhibiting the most significant performance gains. Specifically, DOA-BP achieves an accuracy of 95.78% and an F1-score of 95.77%, slightly outperforming GWO-BP, which reaches 95.63% accuracy and 95.61% F1, by a small but consistent margin. These results confirm the effectiveness of applying DOA for optimizing BPNN parameters, demonstrating that even marginal improvements can be crucial when fine-tuning detection models for sensitive tasks such as T2G communication systems. Although the advantage over other metaheuristic methods is relatively modest, the consistent performance of DOA-BP suggests that it is a reliable optimization approach for improving BPNN models, particularly in distinguishing normal communication behaviors from malicious activities. High performance in this binary classification task reduces the likelihood of false-positives, ensuring that normal T2G communication remains unaffected, which is crucial for avoiding unnecessary safety measures. This capability is essential in railway communication systems, where minimizing the impact on regular operations and ensuring timely, accurate responses to threats are critical.

**Figure 8:**
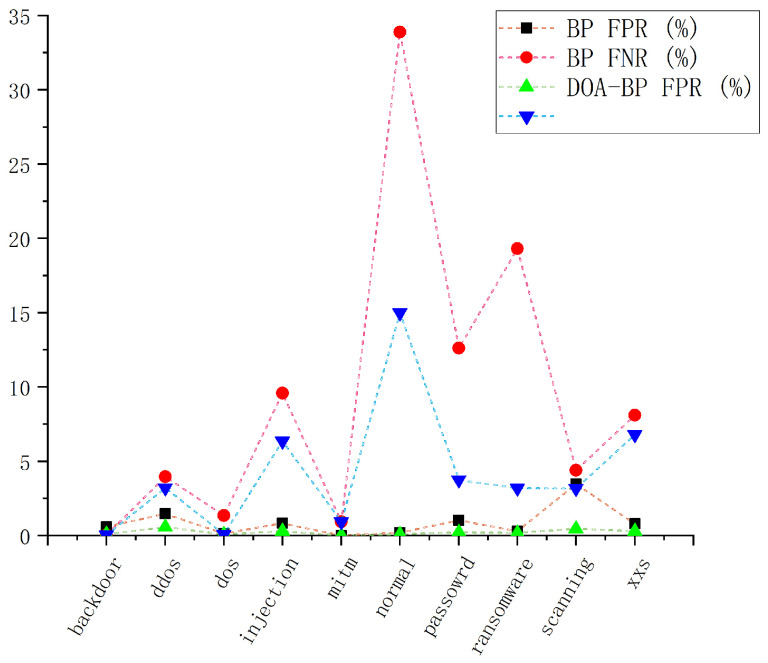
False-positive rate and false-negative rate for multi-class detection.

In T2G intrusion detection scenarios, both false-positive rates (FPR) and false-negative rates (FNR) are critical—a low FPR helps avoid unnecessary alerts, while a low FNR ensures that real threats are not overlooked. As illustrated in Figure 8, DOA-BP and BP show similar performance in terms of FPR, with DOA-BP slightly outperforming BP in most classes, although it occasionally shows higher FPR in specific attack classes. Notably, DOA-BP demonstrates significantly lower FNR compared to BP across key attack classes such as DDoS, DoS, injection, password, ransomware, scanning, and XSS, reflecting its stronger ability to detect genuine attacks and reduce missed detections. For normal traffic, DOA-BP significantly reduces misclassification rates, dropping from 33.88% to 14.99%, indicating that fewer legitimate activities are flagged as threats. However, both models show relatively high misclassification rates in certain classes, with attack classes like password and ransomware exhibiting high FNR, and normal traffic showing considerable misclassification rates. This suggests substantial performance variation between different attack classes, an important observation that warrants further investigation. This balance between minimizing false-positives and reducing missed detections highlights the practical value of DOA-BP in enhancing T2G intrusion detection effectiveness.

### 5.4. Risk-Aware Decision System Results

**Figure 9:**
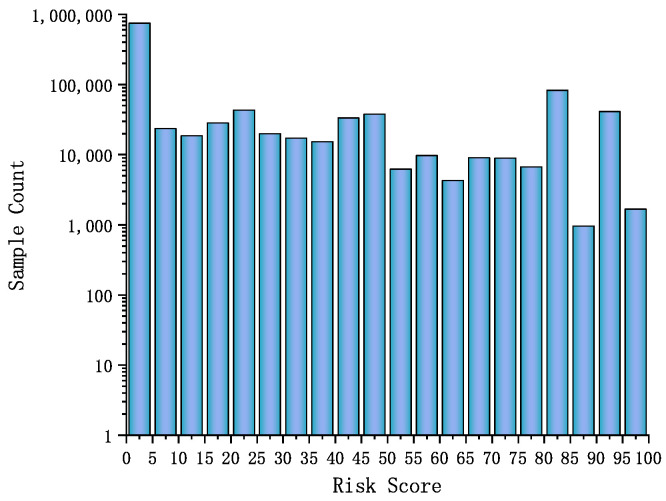
Risk score distributions of samples.

In the second part of the experiment, we evaluated the effectiveness of the proposed intrusion detection and response system in reducing risk levels. Figure 9 shows the distribution of risk scores across all samples, with the X-axis representing risk scores (0–100) and the Y-axis (logarithmic scale) indicating sample counts. The histogram reveals a clear skew toward low-risk values, with a large concentration of samples in the 0–5 range. The logarithmic scale helps better visualize the relatively small number of high-risk samples (scores above 75), which, although rare, require critical security attention. This pattern reflects a typical real-world network scenario, where most traffic is benign, but a minority of events pose significant risks.

**Figure 10:**
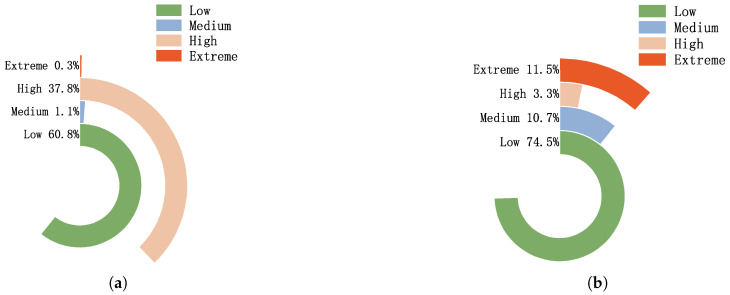
Comparison of risk level distributions under different evaluation mechanisms: (**a**) classification without Bayesian Scoring; (**b**) classification after Bayesian scoring.

Figure 10 compares risk level distributions before and after applying the Bayesian risk scoring module. In subfigure (a), risk levels are assigned based solely on raw detection results, causing over 34% of samples to be misclassified as high risk and less than 2% to fall into medium or extreme categories. Subfigure (b) demonstrates the refined distribution after Bayesian evaluation, where the majority of samples are accurately classified as low risk (74.5%), and high-confidence threats are reassigned to medium and extreme categories (10.7% and 11.5%, respectively), aligning better with the true risk profile. This demonstrates that Bayesian scoring improves precision in risk categorization, enhancing the system’s capability for adaptive defense.

To comprehensively evaluate the proposed security framework, we designed three different security configurations that represent increasing levels of defense sophistication:

1. Rule-based Defense: This configuration uses the DOA-BP detection model to identify attacks. It directly converts anomaly scores into risk scoring and applies basic rule-based responses, without any Bayesian risk scoring or reinforcement learning. This setup represents a typical approach used in traditional security systems.
2. Bayesian Risk Scoring: This configuration builds on the rule-based defense scenario by integrating Bayesian risk scoring for more accurate risk evaluation. The DOA-BP detection model remains the same, but the addition of Bayesian risk scoring leads to better risk identification and more precise risk evaluation compared to the rule-based defense scenario.
3. Full Defense: This configuration extends the Bayesian risk scoring scenario by incorporating an RL-based response module. The Bayesian risk model evaluates risks based on attack type impact, detection confidence, and environmental factors, while the RL module dynamically selects optimal defense actions based on the assessed risk, offering a more adaptive and context-aware defense system.

In each scenario, we measure risk scores at two critical points:

- Pre-Defense: The risk score calculated immediately after an attack is detected, before any defensive action is applied. This reflects the initial severity of the detected threat.
- Post-Defense: The risk score measured after applying the corresponding defense action. This shows how effectively the defense reduces the risk.

**Figure 11:**
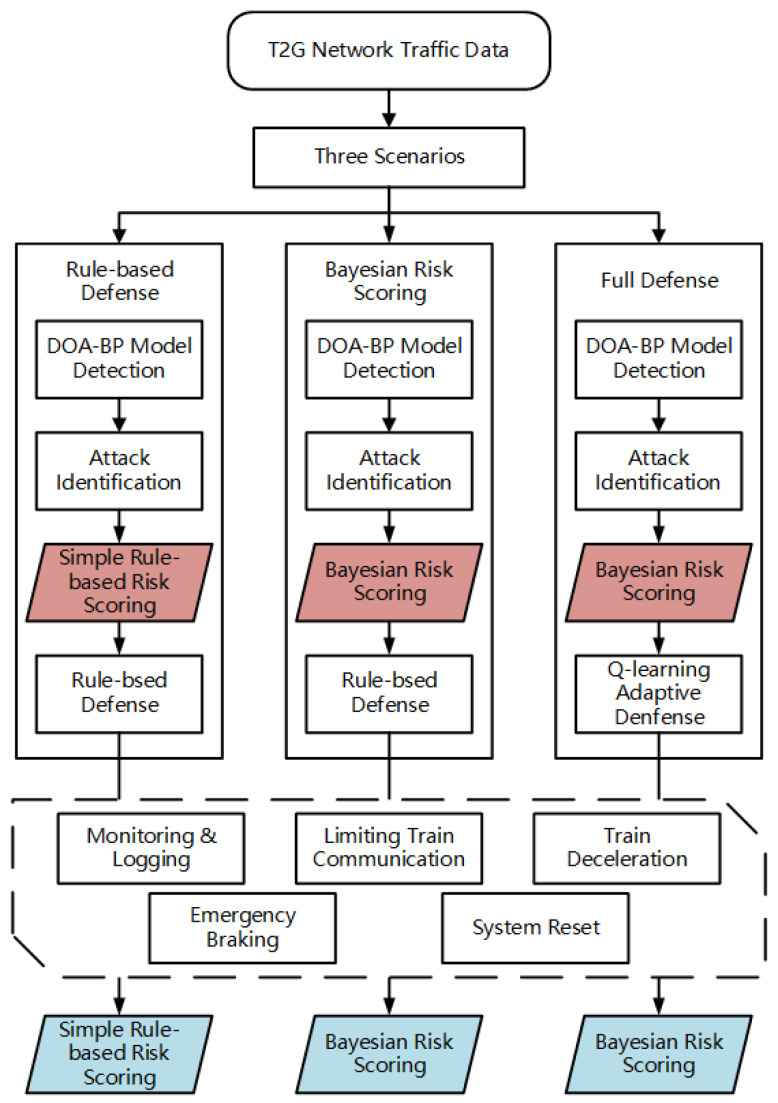
Risk evaluation flow for T2G intrusion detection in three security scenarios. The red diamonds represent pre-defense risk evaluation, while the blue diamonds represent post-defense risk evaluation.

Importantly, while the defensive actions are the same across all scenarios, as defined in Table 4, the method of selecting these actions differs. In the rule-based defense and Bayesian risk scoring scenarios, actions are selected based on fixed rule mappings from risk levels (e.g., low, medium, high, critical); in the Full Defense scenario, the RL module dynamically determines the optimal action based on a broader context. Figure 11 illustrates the flow of risk scoring across the three scenarios, highlighting the pre-defense and post-defense risk evaluation stages.

**Figure 12:**
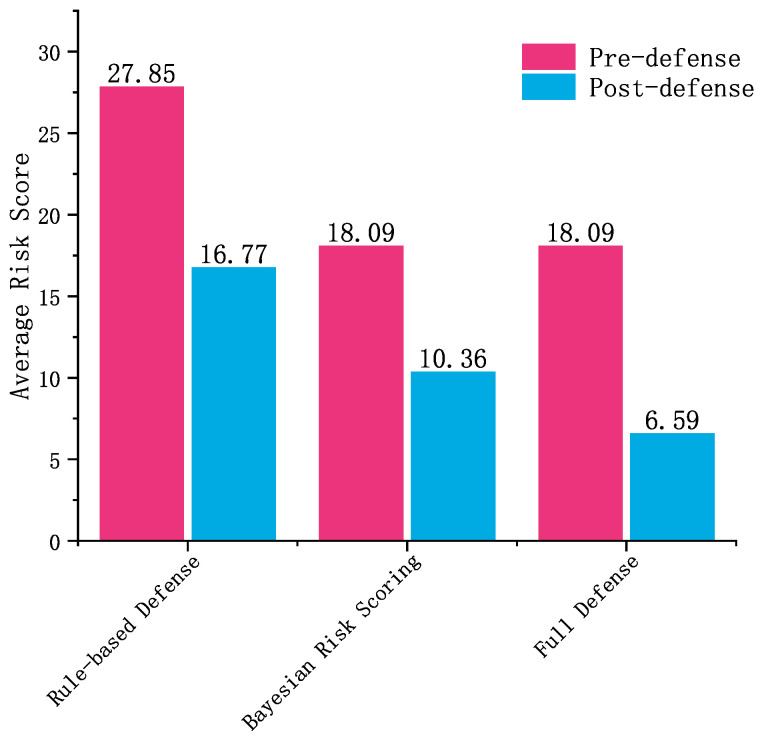
Comparison of risk scores of three security scenarios before and after defense.

Figure 12 compares the average risk reduction results for the three security configurations. To ensure robustness and statistical reliability, we conducted 30 independent trials using 10-fold stratified sampling, reporting the mean risk reductions along with standard deviations and confidence intervals.

In Figure 12, the rule-based defense scenario exhibits a higher pre-defense risk score (mean = 27.85) due to its basic rule-based risk scoring, which may not effectively differentiate between attack types in T2G communication systems. After applying rule-based responses, the average risk is reduced by 39.79% (SD = 0.03). In contrast, both the Bayesian risk scoring and Full Defense scenarios achieve lower pre-defense risk scores (mean = 18.09) by utilizing Bayesian risk scoring, which provides a more accurate evaluation of risk in the context of T2G systems. This method accounts for attack severity, detection confidence, and environmental context, leading to more precise threat identification and better decision-making. The Full Defense scenario, which incorporates both Bayesian risk scoring and reinforcement learning-based adaptive defense, achieves a significant 63.56% risk reduction (SD = 1.60). This demonstrates the value of dynamic defense actions selected based on real-time risk scoring, which is crucial for the timely and effective mitigation of threats in railway communication systems. Additionally, statistical tests confirm that the Full Defense scenario outperforms both the rule-based defense and Bayesian risk scoring configurations, with *p*-values < $1\times 10^{-20}$ in *t*-tests, indicating strong statistical significance.

## 6. Conclusions and Future Work

This research proposes an intelligent security defense framework intended to address key cybersecurity challenges of T2G communications systems, as highlighted in the earlier threat analysis, through three complementary components. The DOA-BPNN intrusion detection model specifically targets network-based attacks identified in the T2G threat model, such as DDoS attacks and unauthorized access attempts that threaten train communication systems. Using the TON_IoT dataset as a baseline, we applied feature selection techniques to extract highly correlated features, reducing dimensionality and redundancy. The Dream Optimization Algorithm was employed to optimize the structure and weights of the BP neural network, resulting in enhanced training stability and detection performance. The Bayesian risk scoring mechanism provides critical context for T2G systems by translating detected anomalies into quantifiable metrics, producing standardized risk scores and level labels that enable operators to understand the potential impact of security incidents on railway operations. By incorporating contextual and probabilistic factors, this mechanism enhances the interpretability of anomaly severity and supports more flexible downstream decision-making. The Q-learning response system offers automated defense selection that is particularly suited to T2G environments, where attack scenarios can rapidly evolve and manual intervention may be delayed, dynamically selecting defense actions that balance risk mitigation and resource efficiency. While using a general IoT dataset for validation, the attack patterns closely resemble those threatening T2G systems, making our framework’s performance relevant to railway communication security. Comparative experiments demonstrated improved results in both binary and multi-class classification tasks, with the Full Defense configuration achieving an average risk score reduction of 63.56%, demonstrating strong adaptability and potential for deployment in high-risk T2G communication scenarios.

Despite the system’s excellent performance in simulation environments, several issues require further exploration. Certain attack types (e.g., ransomware, XSS) still face class imbalance, which can be addressed in future work through data augmentation techniques such as SMOTE or GAN-based synthesis. Additionally, evaluating key operational metrics—such as CPU utilization, response latency, and detection rate—in actual railway operating environments is necessary. A particularly important direction for future research is to apply and validate our framework using railway-specific datasets that contain T2G communication patterns and actual attack samples from railway networks. This will provide more direct evidence of the framework’s adaptability to real-world T2G security challenges and strengthen its practical relevance to railway cybersecurity. Further optimization of the reinforcement learning module is required to improve its learning efficiency and decision-making strategies. Future research should streamline the model for lightweight deployment and assess its scalability and performance across other railway control subsystems. 

## Figures and Tables

**Table 1:** Data structure of TON_IoT dataset.

Class	Dataset Records		
	**Training Set**	**Testing Set**	**Evaluation Set**
Backdoor	45,526	11,382	14,227
DDoS	552,385	138,097	172,620
DoS	302,429	75,608	94,509
Injection	405,582	101,397	126,744
Mitm	94,259	23,565	29,456
Normal	142,710	35,678	44,597
Password	153,983	38,497	48,119
Ransomware	65,233	16,309	20,385
Scanning	639,757	159,940	199,924
Xss	188,960	47,240	59,050

**Table 2:** List of selected features.

No.	Feature Name	No.	Feature Name
1	src_ip	11	src_pkts
2	src_port	12	src_ip_bytes
3	dst_ip	13	dst_pkts
4	dst_port	14	dst_ip_bytes
5	proto	15	dns_qclass
6	duration	16	dns_qtype
7	src_bytes	17	dns_rcode
8	dst_bytes	18	http_request_body_len
9	conn_state	19	http_response_body_len
10	missed_state	20	http_status_code

**Table 3:** Risk level threshold classification.

Risk Level	Range
Low	[0, 25]
Medium	(25, 50]
High	(50, 75]
Extreme	(75, 100]

**Table 4:** Descriptions of response actions.

Decision	Description
Monitoring and Logging	Record and monitor train status and anomalies to detect potential threats in real-time
Limiting Train Communication	Limit the transmission rate of non-critical communications to prioritize train device data
Train Deceleration	Automatically initiate deceleration to ensure smooth operation during an attack
Emergency Braking	Trigger emergency braking to quickly stop the train in case of a severe threat
System Reset	Performs a secure reboot or restores the train device to a safe state

**Table 5:** Experimental setup.

Processor	Intel Core i7 (2.30 GHz, 11th Generation)
Operating System	Windows 10
RAM	32 GB
Language	Python
Matlab Version	Matlab R2022a
GPU	NVIDIA GeForce RTX 3060
IDE	Jupyter Notebook
Numpy	2.2.5
PyTorch	2.7.0
Pandas	2.2.3
Scikit-learn	1.6.1
Batch Size	1024
Population Size	20
Maximum Iterations	60
Training Epochs	80
Hidden Layer Structure	[128, 64]
Learning Rate Scheduler	Cosine Annealing
Initial Learning Rate	0.001
Minimum Learning Rate	$1\times 10^{-6}$

## Data Availability

The data presented in this study are available in the public domain. These data were derived from the TON_IoT dataset, which is openly accessible at https://research.unsw.edu.au/projects/toniot-datasets (accessed on 18 May 2025).

## References

[1] International Union of Railways (2024). Railway Statistics Synopsis.

[2] (2018). Electronic Railway Equipment—Train Communication Network (TCN)—Part 2-6: On-board to Ground Communication.

[3] Moustafa N. (2021). A new distributed architecture for evaluating AI-based security systems at the edge: Network TON_IoT datasets. Sustain. Cities Soc..

[4] Booij T.M., Chiscop I., Meeuwissen E., Moustafa N., Den Hartog F.T. (2021). ToN_IoT: The role of heterogeneity and the need for standardization of features and attack types in IoT network intrusion data sets. IEEE Internet Things J..

[5] Alsaedi A., Moustafa N., Tari Z., Mahmood A., Anwar A. (2020). TON_IoT telemetry dataset: A new generation dataset of IoT and IIoT for data-driven intrusion detection systems. IEEE Access.

[6] Lang Y., Gao Y. (2025). Dream Optimization Algorithm (DOA): A novel metaheuristic optimization algorithm inspired by human dreams and its applications to real-world engineering problems. Comput. Methods Appl. Mech. Eng..

[7] Jain A.K., Mao J., Mohiuddin K.M. (1996). Artificial neural networks: A tutorial. Computer.

[8] Bonafede C.E., Giudici P. (2007). Bayesian networks for enterprise risk assessment. Phys. A Stat. Mech. Its Appl..

[9] Arulkumaran K., Deisenroth M.P., Brundage M., Bharath A.A. (2017). Deep reinforcement learning: A brief survey. IEEE Signal Process. Mag..

[10] Gao B., Bu B., Zhang W., Li X. (2021). An intrusion detection method based on machine learning and state observer for train-ground communication systems. IEEE Trans. Intell. Transp. Syst..

[11] Song Y., Bu B., Zhu L. (2020). A novel intrusion detection model using a fusion of network and device states for communication-based train control systems. Electronics.

[12] Lu H., Zhao Y., Song Y., Yang Y., He G., Yu H., Ren Y. (2024). A transfer learning-based intrusion detection system for zero-day attack in communication-based train control system. Clust. Comput..

[13] Yin B., Bu B., Gao B., Li Q. A hybrid intrusion detection method using improved stacking ensemble algorithm and false positive elimination strategy for CBTC. Proceedings of the 2022 IEEE 25th International Conference on Intelligent Transportation Systems (ITSC).

[14] Li W., Yan Z., He R., Zong L., Zhang F., Zhan Y. A novel machine learning based intrusion detection method for 5G empowered CBTC systems. Proceedings of the 2022 International Wireless Communications and Mobile Computing (IWCMC).

[15] Kong X.Y., Yang G.H. (2022). An intrusion detection method based on self-generated coding technology for stealthy false data injection attacks in train-ground communication systems. IEEE Trans. Ind. Electron..

[16] Fakhereldine A. (2025). Security of Communication-Based Train Control Systems. Ph.D. Thesis.

[17] Chowdhury R., Sen S., Goswami A., Purkait S., Saha B. (2023). An implementation of bi-phase network intrusion detection system by using real-time traffic analysis. Expert Syst. Appl..

[18] Sheikhi S. (2021). An effective fake news detection method using WOA-xgbTree algorithm and content-based features. Appl. Soft Comput..

[19] Jiang H., He Z., Ye G., Zhang H. (2020). Network intrusion detection based on PSO-XGBoost model. IEEE Access.

[20] Hsu C.M., Azhari M.Z., Hsieh H.Y., Prakosa S.W., Leu J.S. (2021). Robust network intrusion detection scheme using long-short term memory based convolutional neural networks. Mob. Netw. Appl..

[21] Lv L., Wang W., Zhang Z., Liu X. (2020). A novel intrusion detection system based on an optimal hybrid kernel extreme learning machine. Knowl.-Based Syst..

[22] Liu T., Yao J., Sun Q. Intrusion detection algorithm of EPSO combined with BP neural network. Proceedings of the 2020 International Conference on Intelligent Transportation, Big Data & Smart City (ICITBS).

[23] Zhao J., Jing X., Yan Z., Pedrycz W. (2021). Network traffic classification for data fusion: A survey. Inf. Fusion.

[24] Abbasi M., Shahraki A., Taherkordi A. (2021). Deep learning for network traffic monitoring and analysis (NTMA): A survey. Comput. Commun..

[25] Attique D., Wang H., Wang P. (2022). Fog-assisted deep-learning-empowered intrusion detection system for RPL-based resource-constrained smart industries. Sensors.

[26] Azimjonov J., Kim T. (2024). Stochastic gradient descent classifier-based lightweight intrusion detection systems using the efficient feature subsets of datasets. Expert Syst. Appl..

[27] Tavallaee M., Bagheri E., Lu W., Ghorbani A.A. A detailed analysis of the KDD CUP 99 data set. Proceedings of the 2009 IEEE Symposium on Computational Intelligence for Security and Defense Applications.

[28] Moustafa N., Turnbull B., Choo K.K.R. (2018). An ensemble intrusion detection technique based on proposed statistical flow features for protecting network traffic of internet of things. IEEE Internet Things J..

[29] Moustafa N., Slay J. UNSW-NB15: A comprehensive data set for network intrusion detection systems (UNSW-NB15 network data set). Proceedings of the 2015 Military Communications and Information Systems Conference (MilCIS).

[30] Siddique K., Akhtar Z., Khan F.A., Kim Y. (2019). KDD cup 99 data sets: A perspective on the role of data sets in network intrusion detection research. Computer.

[31] Tareq I., Elbagoury B.M., El-Regaily S., El-Horbaty E.S.M. (2022). Analysis of ton-iot, unw-nb15, and edge-iiot datasets using dl in cybersecurity for iot. Appl. Sci..

[32] Yaras S., Dener M. (2024). IoT-based intrusion detection system using new hybrid deep learning algorithm. Electronics.

[33] De Menezes F.S., Liska G.R., Cirillo M.A., Vivanco M.J. (2017). Data classification with binary response through the Boosting algorithm and logistic regression. Expert Syst. Appl..

[34] George P.G., Renjith V. (2021). Evolution of safety and security risk assessment methodologies towards the use of bayesian networks in process industries. Process Saf. Environ. Prot..

[35] Sanders R. (1987). The Pareto principle: Its use and abuse. J. Serv. Mark..

[36] Rainio O., Teuho J., Klén R. (2024). Evaluation metrics and statistical tests for machine learning. Sci. Rep..

[37] Mirjalili S., Lewis A. (2016). The whale optimization algorithm. Adv. Eng. Softw..

[38] Rezaei H., Bozorg-Haddad O., Chu X. (2017). Grey wolf optimization (GWO) algorithm. Advanced Optimization by Nature-Inspired Algorithms.

[39] Mirjalili S. (2016). SCA: A sine cosine algorithm for solving optimization problems. Knowl.-Based Syst..

[40] Wang D., Tan D., Liu L. (2018). Particle swarm optimization algorithm: An overview. Soft Comput..

